# Retrospective Evaluation of Control Measures for Contacts of Patient with Marburg Hemorrhagic Fever

**DOI:** 10.3201/eid1807.101638

**Published:** 2012-07

**Authors:** Aura Timen, Leslie D. Isken, Patricia Willemse, Franchette van den Berkmortel, Marion P.G. Koopmans, Danielle E.C. van Oudheusden, Chantal P. Bleeker-Rovers, Annemarie E. Brouwer, Richard P.T.M. Grol, Marlies E.J.L. Hulscher, Jaap T. van Dissel

**Affiliations:** Author affiliations: National Institute for Public Health and the Environment (RIVM), Bilthoven, the Netherlands (A. Timen, L.D. Isken, M.P.G. Koopmans);; Elkerliek Hospital, Helmond, the Netherlands (P. Willemse);; Atrium Medical Centre, Heerlen, the Netherlands (F. van den Berkmortel);; Public Health Service Brabant-Zuidoost, Helmond (D.E.C. van Oudheusden);; Radboud University Nijmegen Medical Centre, Nijmegen, the Netherlands (C.P. Bleeker-Rovers, R.P.T.M. Grol, M.E.J.L. Hulscher);; Elisabeth Hospital, Tilburg, the Netherlands (A.E. Brouwer);; Leiden University Medical Centre, Leiden, the Netherlands (J.T. van Dissel)

**Keywords:** retrospective evaluation, temperature monitoring, control measures, Marburg hemorrhagic fever, Marburg virus, viruses, contacts, daily life, the Netherlands

## Abstract

After an imported case of Marburg hemorrhagic fever was reported in 2008 in the Netherlands, control measures to prevent transmission were implemented. To evaluate consequences of these measures, we administered a structured questionnaire to 130 contacts classified as either having high-risk or low-risk exposure to body fluids of the case-patient; 77 (59.2%) of 130 contacts responded. A total of 67 (87.0%) of 77 respondents agreed that temperature monitoring and reporting was necessary, significantly more often among high-risk than low-risk contacts (p<0.001). Strict compliance with daily temperature monitoring decreased from 80.5% (62/77) during week 1 to 66.2% (51/77) during week 3. Contacts expressed concern about development of Marburg hemorrhagic fever (58.4%, 45/77) and infecting a family member (40.2%, 31/77). High-risk contacts had significantly higher scores on psychological impact scales (p<0.001) during and after the monitoring period. Public health authorities should specifically address consequences of control measures on the daily life of contacts.

## Abstract 2

Measures had substantial effects on contacts and household members.

In July 2008 in the Netherlands, an imported case of Marburg hemorrhagic fever (MHF) (1) was diagnosed in a person after possible exposure in a bat cave in Uganda. MHF is caused by Marburg virus, which belongs to the family *Filoviridae* (2,3). The main route of transmission is by direct contact with blood or body fluids (4). The virus was discovered in 1967 during a laboratory outbreak in Marburg, Germany (5,6). Apart from this person, since the outbreak in Marburg, MHF has been diagnosed only once outside Africa (7).

Because of the high case-fatality rate and propensity for further transmission, a case of MHF is considered to be a public health emergency of international concern and requires prompt intervention to isolate the case-patient and trace and monitor all contacts for early signs of disease. Persons at risk for contracting MHF caused by prior or ongoing contact with an infected person were identified by means of a public health investigation conducted by public health services. The national outbreak response team issued guidelines for classification of these contacts and control measures, including restrictions on leaving the country.

Imported cases of hemorrhagic fever and other severe diseases with the potential to spread among health care workers and the general population have a small, yet realistic chance of occurring in the Western world, as was the situation with Ebola fever and Lassa fever (8–12). Outbreaks can also originate from other sources, as was the case with Ebola-Reston virus (13). Because there are no alternative interventions, such as vaccination or prophylactic treatment, to protect contacts from acquiring MHF, control measures are aimed at early identification of possible case-patients and isolating them from the rest of the population. However, we do not know how persons exposed to MHF respond when confronted with control measures. To date, the consequences of measures to control outbreaks (e.g., monitoring, quarantine) have only been partially investigated for diseases that are not comparable to MHF from the point of view of routes and risk of transmission, e.g., severe acute respiratory syndrome or influenza (14–17). Evidence is needed to determine the effectiveness of follow-up procedures for MHF contacts.

To evaluate the consequences and the psychological effect of control measures on contacts’ daily life, a retrospective cohort study (including serologic testing) was undertaken among 130 contacts of the person in the Netherlands in 2008 who acquired MHF. Contacts were categorized as high-risk or low-risk on the basis of their exposure history. We describe criteria to optimize the effect of control measures and provide proper care to contacts exposed to a person-to-person transmissible virus with the potential to cause severe disease.

## Methods

This study was determined to be part of the public health response to the imported case of MHF and follow-up of contacts. Therefore, explicit ethical evaluation was not necessary.

### Case-Patient Description

On July 2, 2008, after returning from a visit to Uganda from June 5 through June 28, a 41-year-old woman showed development of chills and high fever. She was admitted to hospital A on July 5. Initially, hemorrhagic fever was not included in the differential diagnosis and she was placed in a general ward among other patients, without specific contact precautions. Because she later showed clinical deterioration, liver failure, and tendency to hemorrhage, hemorrhagic fever was suspected and she was transferred to hospital B on July 7. In hospital B, she was placed in strict isolation in accordance with guidelines prescribed for pathogens belonging to Hazard Group 4 (18). We placed the patient in a single room with negative air pressure ventilation and an anteroom for 2 reasons. First, although evidence for airborne transmission of MHF in humans has not been documented, transmission by aerosols has been demonstrated in animal models (7). Second, the patient was likely to undergo aerosol-generating procedures (e.g., endotracheal intubation) while in the latter stages of illness when viral loads in body fluids were expected to be high.

On July 10, the diagnosis of MHF was confirmed; the next day, the patient died. A complete case history and the public health response have been reported (1).

### Control Measures

The outbreak response team formulated measures for follow-up of contacts considered to be at risk for exposure. Measures were based on preexisting national and international guidelines on management of hemorrhagic fever caused by filoviruses (18–20). The patient was considered to be potentially infectious from the onset of fever (July 2) until death (July 11). The period of monitoring contacts was set at 21 days after the last contact with the patient or patient body fluids. The public health service traced the contacts in the community. The hospital hygienist and occupational physician, and attending physicians were responsible for in-hospital contacts. Contacts were provided with written instructions (Table 1).

**Table 1:** Instructions for contacts of a person with Marburg hemorrhagic fever, by risk contact group, the Netherlands, 2008*

Characteristic	Instruction
You have been assigned to the high-risk category	You have shared the household (or the ward) with the patient. You have cared for the patient in the hospital without wearing PPE. You had or might have had unprotected contact (without PPE) with the blood or body fluids of the patient.
The following restrictions have been imposed on you	Remain in the neighborhood of your home address during the monitoring period of 3 weeks after last possible contact (date). Stay in contact with the health care provider you have been assigned (the public health service, the hospital hygiene specialist, or the occupational medicine specialist). Do not leave the country. Cancel or postpone a holiday trip abroad.
Instructions on control measures during monitoring period	Inform your health care provider if you use temperature-lowering medication. Measure your temperature in the morning and evening. Use your own thermometer (one that is not to be used by others) and write down your temperature accurately. Disinfect the thermometer with 70% alcohol after every use and wash your hands with soap and water. Contact your health care provider daily and provide him or her with information about your health and temperature. If you have a fever (2 consecutive temperature measurements ≥38°C 12 h apart), vomiting, headache, stomach ache, diarrhea, jaundice, or cough, immediately contact your care provider. Stay at home and restrict all contacts with others until further instructions from your health care provider. Only use your own toilet.
You have been assigned to the low-risk category	You cared for the patient (using adequate PPE) while she was admitted to the hospital and in accordance with a strict isolation protocol. You had contact with blood or body fluids of the patient while using effective PPE.
Instructions on control measures during monitoring period	Inform your health care provider if you use temperature-lowering medications. You are strongly advised not to leave the country during the monitoring period of 3 weeks after the last possible contact with the patient or patient body fluids (date). Measure your temperature daily in the morning and evening. Use your own thermometer (one that is not to be used by others) and write down your temperature accurately. Disinfect the thermometer with 70% alcohol after every use and wash your hands with soap and water. If you have a fever (2 consecutive temperature measurements ≥38°C 12 h apart), immediately contact the health care provider you have been assigned (public health service, the hospital hygiene specialist, or the occupational medicine specialist). Stay at home and restrict all contacts with others until further instructions from your care provider. Only use your own toilet.

*PPE, personal protective equipment.

Contacts classified as at high risk for contracting the disease (i.e., had unprotected contact with the patient or her body fluids) were asked to measure their temperature twice a day and report it to the health care provider. Furthermore, they were prohibited from leaving the country and were told to report any intention to leave to the public health authority. Contacts were asked to notify the assigned health care provider immediately if they had fever (body temperature >38°C measured twice at least 12 hours apart) or any abnormal symptoms (e.g., vomiting, headache, abdominal pain, diarrhea, jaundice). Contacts who had handled the patient or her body fluids while carrying out strict isolation measures were perceived to be at low risk for exposure. They were asked to measure their temperature twice a day, but only to report a temperature >38°C. This group was strongly encouraged not to travel abroad.

### Study Population

A retrospective cohort study involving the 130 contacts was conducted by using an online questionnaire. High-risk contacts (n = 64) included household contacts, health care providers at the general practice, those involved in patient care at hospital A (nurses, physicians, and laboratory workers), patients sharing the same hospital ward in hospital A, and cleaning staff. Low-risk contacts (n = 66) included health care and laboratory workers at hospital B who had all taken appropriate personal protective measures. Data were collected from December 2008 through February 2009, which was 5–7 months after possible exposure. At the same time, serologic testing was conducted to assess asymptomatic transmission.

### Variables and Instruments

#### Questionnaire

We recorded personal characteristics of respondents and any symptoms during the monitoring period. The questionnaire (62 questions) addressed understanding of control measures, clarity of instructions, reported compliance with measures, and perceived interference with daily life (e.g., restrictions on social life, difficulties with control measures, extra costs incurred, and fear of becoming infected). To develop questions regarding interference and reported compliance, we drew from the current literature on the effect of restrictive measures during outbreaks (14,15,21).

Questions regarding clarity of instructions and understanding measures were based on parts of the Consumer Quality Index Instrument of the Netherlands Institute for Health Services Research (Rotterdam, the Netherlands), which was derived from the Consumer Assessment of Healthcare Providers and Systems Survey developed and funded by the US Agency for Health Care Research and Quality (Rockville, MD, USA) (22). Depending on the type of question, respondents were asked to answer either yes or no questions, or to choose an option on Likert scales of 1–5 (1, completely disagree; 2, disagree; 3, neutral; 4, agree; and 5, strongly agree or 1, never; 2, seldom; 3, sometimes; 4, often; 5, always).

#### Impact of Event Scale

To evaluate stress levels, we used the Revised Impact of Event Scale (IES-R; Dutch version), a 22-item international instrument designed to measure situations in life that are perceived as stressful (23). The instrument was divided into 3 subscales: intrusion (e.g., recurrent thoughts); avoidance (e.g., avoiding reminders of the event); and hyperarousal (e.g., concentration and sleeping problems). Each subscale contained questions derived from the Diagnostic and Statistical Manual of Mental Disorders criteria (4th edition) for posttraumatic stress disorder (24). Items were scored on a Likert scale of 0–4 (0, not at all; 1, a little bit; 3, quite a bit; 4, extremely), which added up to a maximum score of 88. A score >20 was considered an indicator of posttraumatic stress disorder (14,15). We assessed recalled stress during the monitoring period (IES during) and persisting stress during the 7 days before the completion of the questionnaire (IES after).

### Statistical Analysis

Data were analyzed by using SPSS version 18 (SPSS Inc., Chicago, IL, USA). Descriptive statistics were calculated. Means and SDs were calculated for answers given on the Likert scale. Results were stratified by type of exposure risk (i.e., high or low risk for disease transmission). Differences in proportions were calculated by using the χ^2 ^test. Differences in means were calculating by using the Student *t *test. A p value <0.05 was considered significant.

We constructed overall scales. The compliance with measures scale (Cronbach α 0.71) included 4 questions regarding temperature monitoring, temperature reporting, and prohibition on travel. The interference scale (Cronbach α 0.64) included 11 questions on perceived restrictions on social life, difficulties with control measures, extra costs, and anxiety of contacts and their families. The clarity of instructions scale (Cronbach α 0.82) included 5 questions on explicitness, completeness, unambiguity, confusion, and redundancy of the provided instructions. The overall scale for understanding of measures (items regarding awareness of the measures and their rationale) proved invalid and was not used in further analysis.

To determine which variables influenced stress levels during the monitoring period, we developed a linear regression model by using the IES during monitoring as a dependent variable and personal characteristics (sex, education, age), risk level, clarity of instructions scale, compliance scale, and interference scale as independent variables. The same model was constructed by using the IES after the monitoring period as the dependent variable.

### Serologic Testing

Serologic testing was performed by using an immunofluorescence antibody (IFA) assay with blood samples collected from contacts 5–7 months after the monitoring period. IFA test slides were prepared at the Bernhard Nocht Institute for Tropical Medicine (Hamburg, Germany) by using the patient strain. Laboratory methods have been reported (1).

## Results

### Response

Of 130 eligible participants, 78 (60.0%) completed the questionnaire. One person provided systematically inconsistent answers and was excluded, which left 77 respondents (59.2%) for statistical analysis. The response rate was 70.3% (45/64) in the high-risk group and 48.5% (32/66) in the low-risk group (Table 2). Of the 77 respondents, 46 (59.7%) were female, 44 (57.1%) did not have children in the household, and 11 (14.3%) lived alone. Mean age was 38 years in the high-risk group and 43 years in the low-risk group (this difference was not significant). The high-risk group had more women (33/45, 73.3%) than the low-risk group (13/32, 40.6%) (p = 0.004) and had a lower education level (Table 2). Respondents were comparable to nonrespondents with respect to sex (M:F ratio 40.0%:60.0% vs. 42.0%:58.0%). During the monitoring period, nonspecific symptoms (those commonly occurring during the prodromal phase of hemorrhagic fever syndromes, e.g., headache, malaise, fatigue) (2) developed occasionally in some contacts, but no significant differences were observed between the risk groups.

**Table 2:** Characteristics and self-reported signs and symptoms of persons who had contact with a Marburg hemorrhagic fever patient, the Netherlands, 2008*

Characteristic	High-risk contact, n = 45, no. (%)	Low-risk contact, n = 32, no. (%)	p value†
Sex			**0.004**
F	33 (73.0)	13 (40.0)	
M	12 (37.0)	19 (60.0)	
Age, y			0.127
<25	13 (29.0)	2 (6.2)	
26–35	9 (21.0)	8 (25.2)	
36–45	10 (22.0)	10 (31.2)	
46–55	10 (22.0)	11 (34.3)	
56–65	3 (6.0)	1 (3.1)	
Education			**0.002**
Secondary	8 (18.0)	1 (3.0)	
Vocational	17 (37.0)	4 (12.0)	
Higher professional	15 (34.0)	22 (70.0)	
University	5 (11.0)	5 (15.0)	
Health status			**0.047**
Excellent	11 (24.0)	15 (47.0)	
Very good	19 (42.0)	6 (18.7)	
Good	15 (34.0)	11 (34.3)	
Reported conditions during monitoring period			
Temperature >38°C	2 (4.4)	2 (6.2)	0.901
Headache	17 (37.7)	8 (25.0)	0.064
Myalgia	5 (11.1)	1 (3.1)	0.407
Malaise	13 (29.0)	10 (31.2)	0.513
Nausea	6 (13.3)	2 (6.2)	0.429
Abdominal pain	6 (13.3)	3 (9.3)	0.482
Fatigue	11 (24.0)	6 (18.5)	0.222
Vomiting	1 (4.5)	0	0.399
Diarrhea	6 (13.3)	1 (3.1)	0.247

*High-risk contact, unprotected contact with the patient or her body fluids; low-risk contact, contact with the patient or her body fluids while following strict isolation measures. **Boldface** indicates statistical significance (p<0.05). †By χ^2^ test.

### Understanding Measures

Of the 77 respondents, 60 (77.9%) were aware of the request not to leave the country, and 17 (22.1%) chose the neutral option. Of 60 contacts aware of the request, 54 (90.0%) in the high-risk group agreed with this request (p = 0.04). Reasons given for negative answers were no direct contact with the patient and thus no risk (n = 2), imperative reasons to leave the country such as illness in the family (n = 2), travel restrictions were meant for others, not for me (n = 1), and borders do not stop diseases (n = 1). All respondents were aware of the need to measure temperature twice a day, 67 (87.0%) agreed with the necessity of measuring and reporting temperature, and 7 (9.1%) had no opinion. Not feeling ill was the reason for disagreement in the remaining 3 (3.8%) respondents. Only 58 (75.3%) of the 77 respondents correctly identified the rationale of temperature monitoring. Respondents in the high-risk group agreed more often than their low-risk counterparts with the necessity of daily measuring and reporting for temperature (mean ± SD of agreement in the high-risk group 4.7 ± 1.0 vs. 1.6 ± 1.3 in the low-risk group) (p<0.001). Written instructions with detailed information on the control measures were received by 61 (79.2%) of 77 respondents. Of these 61 respondents, 45 (73.7%) found this information to be clear, 40 (65.6%) complete, 37 (60.6%) unequivocal, 4 (6.5%) confusing, and 3 (5.0%) redundant. There were no significant differences between the risk groups.

### Compliance with Measures

Of 45 respondents who were prohibited from traveling (high-risk group), 17 (37.7%) had planned a holiday trip abroad, of whom 12 (70.6%) cancelled the holiday trip during the surveillance period, and 1 (5.9%) postponed the trip. Two (11.8%) high-risk contacts were already abroad when the diagnosis in the index case was made and 2 (11.8%) other contacts left the country a few days before the end of the surveillance period, despite prohibition on travel. Risk management in these persons, including communication with government authorities in countries to which they traveled, has been reported (1). Temperature was monitored twice a day by 62 (80.5%) of 77 persons, and 75 (97.4%) of 77 had a thermometer at their immediate disposal. Strict compliance with daily temperature monitoring decreased from 80.5% (62/77) in week 1 to 66.2% (51/77) in week 3. Differences per risk group per week in reported compliance with temperature monitoring and reporting are shown in Table 3.

**Table 3:** Compliance with temperature monitoring and reporting in persons who had contact with a person with Marburg hemorrhagic fever, the Netherlands, 2008*

Variable	High-risk group score, mean (SD)	Low-risk group score, mean (SD)	p value†
Temperature monitoring week			
1	4.87 (0.63)	4.25 (1.16)	0.004
2	4.87 (0.63)	3.84 (1.30)	<0.0001
3	4.82 (0.68)	3.34 (1.54)	<0.0001
Temperature reporting week			
1	4.73 (1.01)	1.56 (1.37)	<0.0001
2	4.73 (1.01)	1.50 (1.34)	<0.0001
3	4.71 (1.01)	1.50 (1.34)	<0.0001

*Answers were given on a 5-point Likert scale, according to the following categories: 1, never; 2, seldom; 3, sometimes; 4, often; 5, always. High-risk contact, unprotected contact with the patient or her body fluids; low-risk contact, contact with the patient or her body fluids while following strict isolation measures. †By 2-sample Student *t *test assuming unequal variances. Because of the relatively large sample sizes of 45 and 32 and data that consisted of scores from 1 through 5 with the indicated SDs, this test is justified by the central limit theorem and because the t distribution with 75 = 45 + 32 – 2 df is almost identical with that of a normal distribution. All p values were statistically significant (p<0.05).

### Interference of Measures with Daily Life

The prohibition on leaving the country was perceived as difficult by 18 (23.4%) of 77 respondents; 11 (14.3%) perceived serious restrictions on their social life, and 24 (31.2%) reported feeling stressed on a regular basis during monitoring. Daily temperature monitoring was believed to be troublesome by 25 (32.5%) of 77. Extra costs were involved for 19 (24.6%) of the contacts. The questionnaire did not ask for specification of costs incurred. Of 47 health care workers among respondents, 13 (27.6%) intensified adherence to infection prevention guidelines at work during the monitoring period (mean ± SD intensified adherence in the high-risk group 1.3 ± 0.4 vs. 1.03 ± 0.2 in the low-risk group) (p = 0.006). Sustained and intensified adherence to infection prevention at work after the monitoring period ended was reported by 10 (21.3%) of 47.

Being identified as a contact caused anxiety in respondents, as reflected in the high percentage who were afraid of contracting MHF (45/77, 58.4%), those who were concerned about infecting other members of their household (31/77, 40.3%), or those who were afraid that a colleague might have been unknowingly infected by the index patient (35/77, 45.5%). Of 77 respondents, 28 (36.4%) reported that their family members were disturbed by control measures. Furthermore, 31 (40.3%) of 77 reported that their family members expressed anxiety about becoming infected, and 25 (32.5%) of 77 reported that their partners were disturbed by restrictive control measures. The results on the overall interference scale were compared and showed that control measures caused more interference with daily life (lower mean ± SD) in the high-risk group (mean ± SD 1.5 ± 0.3 vs. 1.8 ± 0.2; p<0.001) than in the low-risk group.

### Impact of Event Scale

Means were calculated on the basis of the answers on the IES and subscale domains (intrusion, avoidance, and hyperarousal). Overall psychological distress was measured by the mean ± SD score on the IES during monitoring (2.8 ± 2.6, range 0.2–10) and 5–7 months after monitoring 0.9 (0.9 ± 1.7, range 0–9.5; p<0.001). Despite the low overall mean score, high individual scores were observed (Figure). On the subscale domains, the mean scores were higher during the monitoring period than after it, and the highest scores were reported in the high-risk group.

**Figure:**
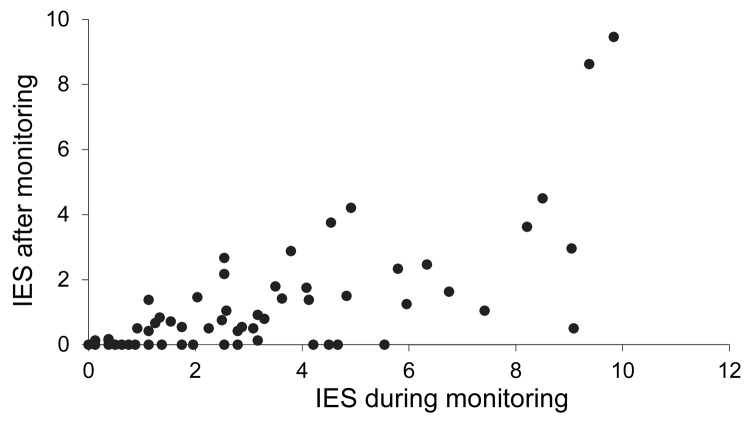
Distribution of individual scores on the impact of event scale (IES) during and after (a 7-day period before completion of a questionnaire) the monitoring period among contacts of the person with Marburg hemorrhagic fever, the Netherlands, 2008. Each circle indicates 1 person. A higher score indicates a higher level of stress.

In the linear regression model, the score on the interference scales was a predictor of the score of the IES during monitoring, which measured the psychological effect of this event (p<0.001). After monitoring ended, the remaining psychological effect measured by the IES score was influenced by the degree of interference (p<0.001) and level of education (p = 0.015); persons with a lower education level reported more psychological consequences (Table 4).

**Table 4:** Linear regression model of scores on Impact of Event Scale during and after the monitoring period in persons who had contact with a person with Marburg hemorrhagic fever, the Netherlands*

Variable	IES score during monitoring			IES score after monitoring	
	B (SE)	p value		B (SE)	p value
Constant	4.73 (0.75)			2.62 (0.59)	
Sex	0.11 (0.14)	0.446		0.10 (0.11)	0.349
Education	−0.14 (0.08)	0.103		−0.17 (0.06)	**0.015**
Age	0.02 (0.05)	0.714		0.04 (0.04)	0.372
Risk category	−0.06 (0.16)	0.742		−0.06 (0.13)	0.663
Clarity of instructions scale†	−0.02 (0.08)	0.864		−0.03 (0.06)	0.678
Compliance scale‡	0.10 (0.08)	0.296		−0.05 (0.06)	0.469
Interference scale§	−2.19 (0.25)	**<0.001**		−1.08 (0.19)	**<0.001**
R^2^	0.601			0.437	

*IES, impact of event scale; B, estimate of regression coefficient (a negative estimate indicates a negative association of the independent variable with the dependent variable); R^2^, proportion of variance in the dependent variable accounted for by the model. **Boldface** indicates statistical significance (p<0.05). †Explicitness, completeness, unambiguity, confusion, and redundancy. ‡Twice a day temperature monitoring, temperature reporting, canceling trip, and postponing trip. §Difficulties with monitoring, extra costs, fear of becoming infected, fear of infecting household members, household members fearing infection, fear that colleagues might be infected, restrictions in social life, tensions caused by the measures, tensions experienced by partners, and tensions experienced by children in the household.

### Serologic Testing

IFA serologic testing was conducted in 85 (65.4%) of 130 participants, which represented 50 (78.1%) of 64 in the high-risk group and 35 (53.0%) of 66 in the low-risk group. All serum samples were negative for IgG and IgM against Marburg virus.

## Discussion

Being at risk for acquiring MHF had a measurable psychological effect and personal consequences (such as restrictions on social life) on respondents in both risk contact groups. We will discuss implications for persons who have to adhere to control measures, discuss the psychological effect of these measures, and reflect on the prerequisites for applying control measures.

Compliance with control measures was reported as difficult to include in daily life, a finding consistent with reports concerning a patient with imported Lassa hemorrhagic fever (12,25). Some practical problems need to be addressed to facilitate compliance with temperature monitoring and prohibitions on leaving the country. Problems were related to comprehensiveness of measures. Some respondents had already paid for a holiday trip and subsequently had to cancel it. Increased costs that persons incurred imply that compensation policies (e.g., insurance) should be explained or made available in preparedness plans (26). Practical constraints related to temperature monitoring should be averted by making use of online registration systems and modern technologies to contact persons and facilitate communication with health care providers.

Although temperature monitoring remains the primary method of detecting onset of fever and enabling follow-up of contacts during the incubation period (6), appropriateness of once a day vs. twice a day monitoring is a disputed issue. In accordance with guidelines in the Netherlands (19) and the Centers for Disease Control and Prevention Interim Guidance (20), we implemented twice a day temperature monitoring. In outbreak situations, once a day monitoring is usually followed. Although twice a day monitoring is not an evidence-based recommendation, we applied it to be able to discriminate between a single episode of fever and sustained fever needing follow-up. Given our experience, twice a day monitoring is appropriate for management of high-risk contacts of a single case-patient or few patients with imported cases, but might not be feasible in every outbreak situation.

Our study shows that control measures had a substantial psychological effect on contacts and members of their household. By quantifying the recalled level of stress at 2 time points, we demonstrated that stress-related complaints persisted for a longer period, thus enlarging the body of evidence built on the basis of previous outbreaks (14,15,27–30). We found the interference scale score to be the main predictor of perceived stress, suggesting that the disturbance experienced in daily life determines the magnitude of the psychological effect.

In the light of these findings, what should the prerequisites be for applying control measures? Public health authorities in charge of crisis management need to be aware of difficulties and stress-related issues experienced by persons to whom the measures apply and address them systematically in the crisis guidelines. Apart from overcoming practical barriers, public health authorities should address the psychosocial well-being of persons being monitored and their family members. Although health care in contacts is focused mostly on early identification of somatic symptoms, concerns, anxiety, and stress experienced by those involved are also crucial. Guidelines should emphasize the need for individual support to assess and manage stress and to address questions about personal risks (29).

Finally, occasional incidents of noncompliance with the prohibition on leaving the country have policy implications, which make intergovernmental collaboration necessary. Restricting freedom to travel during the incubation period of persons with high-risk exposure to a transmissible pathogen obliged by international health regulations to be reported requires effective legislation, an issue that should ideally be dealt with before such an incident occurs. Although voluntary compliance, based on effective communication and trust in the authorities, is the most appropriate approach (31), should compulsory means be necessary in the Netherlands, emergency legal provisions are now in place to impose travel restrictions and to monitor the health status of contacts who have been exposed to certain pathogens.

Despite being sent reminders, the response rate of participants was only 60.0%. This limits interpretation of results because motives for noncompliance remain unknown. As in most retrospective studies, we also need to acknowledge the chance of recall bias, which might influence recollection of experiences. Another limitation was inherent to the study design, which required answers only from contacts. Thus, consequences of control measures on members of the household could only be assessed indirectly. Despite the limitations of IFA (32), serologic findings strongly suggest that none of the contacts who provided a blood sample had become asymptomatically infected.

The strengths of this study are 1) systematic evaluation of compliance with measures and interference of measures with daily life, and 2) quantification of psychological effects of these measures during and after this major event, which had received much attention in the media. We reinforce the hypothesis that psychological symptoms may persist longer. Public health authorities need to be aware of the immediate and long-term effects that measures can have on persons being monitored.
